# “When in Rome, Do as the Romans Do”: Cultural Barriers to Being Agile in Distributed Teams

**DOI:** 10.1007/978-3-030-49392-9_10

**Published:** 2020-05-06

**Authors:** Darja Šmite, Javier Gonzalez-Huerta, Nils Brede Moe

**Affiliations:** 6 University of Oslo, Oslo, Norway; 7 Monash University, Clayton, VIC Australia; 8 IT University of Copenhagen, Copenhagen, Denmark; 9 University of British Columbia, Vancouver, BC Canada; 10 Blekinge Institute of Technology, Karlskrona, Sweden; 11 SINTEF ICT, Trondheim, Norway

**Keywords:** Culture, Cultural differences, Agile, Distributed development, Distributed agile teams

## Abstract

With the growing interest of adopting agile methods in offshored process, many companies realized that the use of agile methods and practices in companies located outside the location of early adopters of agile methods may be challenging. India, the main destination of offshoring contracts, have received particular attention, due to the big cultural differences. Critical analysis of related studies suggests that impeding behaviors are mostly rooted in the hierarchical culture of Indian organizations and related management behavior of command-and-control. But what happens in distributed projects with a more empowering onshore management? In this paper, we present the findings from a multiple-case study of DevOps teams with members from a mature agile company located in Sweden and a more hierarchical offshore vendor from India. Based on two focus groups we list culturally different behaviors of offshore engineers that were reported to impede agile ways of working. Furthermore, we report the findings from surveying 36 offshore team members from five DevOps teams regarding their likely behavior in situations reported to be problematic. Our findings confirm a number of previously reported behaviors rooted in cultural differences that impede the adoption of agile ways of working when collaborating with offshore engineers. At the same time, our survey results suggest that among the five surveyed teams there were teams that succeeded with the cultural integration of the offshore team members. Finally, our findings demonstrate the importance of cultural training especially when onboarding new team members.

## Introduction

The times when software could be designed by a single co-located agile team, are long gone and many agile software development environments have become highly distributed. Software companies often collaborate with engineers from multiple sites of the same company or from sub-contractors. Thus, agile teams might be spread over several time zones and geographic locations, implying that different national and organizational cultures are represented [25]. Such setups increase the complexity of the software development work and challenges emerge [10]. One example of such challenges is when more hierarchical organizations from certain Asian countries (e.g., India or China) collaborate with self-managing teams from the Nordic countries. Self-managing agile teams are those given significant authority and responsibility for many aspects of their work, such as planning, scheduling and assigning tasks to members, and making decisions with economic consequences [19]. An interesting question is what happens when teams are set up with a mix of representatives from a mature agile organization and a more hierarchical organization?

The first challenges emerge when crossing organizational boundaries. Several researchers have emphasized the importance of cultural compatibility or fit between the organizational culture and the software development method in use [6, 11], and that companies are likely to encounter difficulties when having incompatibilities. What makes it challenging to reach compatibility is the fact that even in agile organizations you will find several conflicting sub-cultures [13, 20] e.g. when the culture at the agile team level is seen as a threat because it conflicted with existing and established habits of the management. This means that team is a relevant context to study culture.

The next big challenges emerge when crossing national boundaries. Collaboration with Asian vendors is one big trend in many Western companies. Many of these companies report challenges when introducing agile in projects involving offshore engineers, rooted in the cultural differences [1, 5, 10, 15, 27, 30]. In particular, companies from the main offshore destination, India, have focused on rigid process improvement programs as a means of demonstrating organizational capabilities, and therefore often exhibit heavily plan-based culture with corresponding organizational structures and processes [27]. Whether and how to achieve combability between the national culture and agile development methods when creating virtual teams involving team-members with radically different cultural backgrounds has thus become an important research topic. However, some researchers warn that the major differences in norms and values cannot be harmonized, since they derive from deep-seated differences in cultural background, education, and working life [14]. The need to understand how to succeed with the adoption of agile ways of working in globally distributed teams with members from an Indian vendor, motivated us to explore what are the specific cultural barriers, what resulting behaviors impede agility, and whether these behaviors prevail among offshore engineers working in distributed agile teams. Our empirical study therefore addresses the following research questions:

- **RQ1**: What are the cultural barriers impeding agile ways of working in distributed teams with members from a hierarchical culture?
- **RQ2:** What can agile teams do to integrate the offshore members?

In this paper, we report our results from an empirical study of a Swedish company working with offshore engineers from an outsourcing vendor in India. The rest of the paper is organized as follows. Section 2 summarizes the links between cultural differences and the behavior of Asian software engineers, as well as the role of cultural differences when introducing agile ways of working in offshore projects. Section 3 introduces our research methodology and the case company. The results of our study are presented in Sect. 4, followed by a discussion in Sect. 5. Section 6 concludes the paper with a summary of the findings and implications for practice and further research.

## Background and Related Work

Culture is related to the way we give logic to the world and begins at birth with gestures, words, tone of voice, noises, colors, smells, and body contact we experience [18]. Our culture is what is familiar, recognizable, habitual, it is “what goes without saying”, “what is normal”. Yet, culture is a multifaceted concept, and can be attributed to a nation, an organization, a group or even an individual, because it is shaped by one’s social environment [9]. Therefore, culture is a sensitive subject and not the depiction of wooden stereotypes [3]. Yet, common characteristics may exist that distinguish one culture from another [3]. In our work, we focus on the impact of the national culture and the organizational culture on the ways of working.

National cultures and cultural differences have been studied in-depth by several social scientists (e.g. [9] and [7] to name a few). These studies resulted in several overlapping cultural characteristics that are common for representatives of a particular nation. National culture may determine preferred leadership styles and decision-making processes, perceptions of authorities, attitude towards time, need for formalization, preferred communication and interaction styles, business etiquette and motivation tools [9]. Similarly to organizational incompatibilities with the method in use [6, 11], incompatibilities in the national backgrounds and the differences in the ways of working can prove problematic [10, 14]. In fact, the larger the degree of difference in organizations and national cultures, the larger the cultural distance between the parties involved [4]. In the following, we first explain what characterizes agile ways of working and what organizational culture is conducive for successful adoption of agile methodology, and then summarize research studies related to the challenges of introducing agile ways of working in Asia, relevant for our empirical study.

### Agile Ways of Working and Organizational Culture

Agile ways of working stem from a group of methods united by a common philosophy, values, and principles. It emphasizes teamwork and heavily relies on the ability of a software team to self-manage [8, 19]. The principles of self-management and autonomy, central to agile ways of working, put certain demand on the organizational culture, team composition and behavioural norms [8, 21, 28]. Morgan [23] emphasises the importance of teams’ ability to engage in self-learning and drive continuous improvement, and ability to act upon minimum critical specification. van Solingen et al. [26] argue that the prerequisites for improvement and learning are openness and the ability to discuss the underlying problems. Based on two large surveys of agile teams, Williams captures practices essential for teams to be considered agile being related to their ability to satisfy the customer through early, continuous and frequent delivery of valuable, working software; the prerequisite for which is, among others, staffing projects with motivated individuals who are given the needed resources and authority to get their job done [31].

A number of studies investigated the relationship between organizational culture and the use of agile methods [8, 12, 13, 29]. Based on a multi-case study of nine projects Strode et al. [29] found that specific organizational culture factors correlate with effective use of an agile method. Their findings suggest that an organization is more likely to be successful if the organization values feedback and learning; social interaction in the organization is trustful, collaborative, and competent; the project manager acts as a facilitator; the management style is that of leadership and collaboration; the organization values teamwork is flexible and participative and encourages social interaction; the organization enables empowerment of people; the organization is results oriented; leadership in the organization is entrepreneurial, innovative, and risk taking; and the organization is based on loyalty and mutual trust and commitment [29]. Similar findings emerged from studying 58 agile practitioners from 23 organizations in New Zealand and India [8]. Hoda et al. found that the prerequisite for self-organizing agile teams to establish and flourish is senior management support, in terms of providing freedom and establishing an organizational culture of trust. They also suggest that an organization with a strict hierarchical structure is not conducive to self-organizing agile teams, because the hierarchy enforces a lack of openness marked by restricted and indirect lines of communication and feedback, which in turn leads to an environment of fear [8]. Based on a multi-case study, Kautz et al. [13] found that agile development thrives in different organizational cultures, even in hierarchical ones, as long as the 4 core values are present to a significant extent. Furthermore, they argue that while organizational culture has an impact on the way agile development is enacted, in practice it is often the method which is adjusted to the organization. Similarly, Iivari et al. argue that the relationship between an organizational culture and agile ways of working is dynamic and therefore will continuously evolve [12]. This means that time perspective matters and studies on the compatibility between the culture and agile ways of working shall take the dynamic nature of this relationship into account.

Another reason to look at the organizational culture from a time perspective is the staff changes. When adding new people to an already established agile team, it is essential to support the new team members in adapting to the existing teams culture and ways of working, which is especially difficult in virtual setups. In their study of onboarding Portuguese developers into existing Norwegian agile teams [22], Moe et al. conclude that the most important success factor is finding people that matched the culture of the existing teams. Therefore, during an onboarding process, all interviews and visits need to focus on communicating the values and culture and on giving insight into the existing organization’s norms.

### Agile Adoption in Asian Countries

Since, national culture is said to have significant influence on the organizational culture [9] and organizational culture may impact the use and success of agile ways of working [13, 29], there is interest in understanding the use of agile methods and practices in companies located outside the locations of early adopters of agile methods. In particular, researchers and practitioners have wondered about the abilities of the companies and engineers from the Asian region, the primary recipients of offshoring contracts, to adopt the agile ways of working, which are so distinct to their national culture.

To address these questions, a number of studies sought evidence of successful use of agile methods in offshored projects [10, 25]. Some researchers infer the successful adoption from the large number of practices reported as being followed [2, 32]. However, the validity of these studies as well as the research approach are questionable, because high level of commitment to the use of agile practices can be explained by the readiness to accept the established rules in hierarchical (i.e., high power distance) cultures, as found, for example, in a study of agile adoption in Malaysia [1]. Other research studies tried to improve the understanding of what specifically impedes the adoption of agile ways of working in Asian cultures [1, 5, 8, 14, 15, 30] and how to succeed [8, 25]. In Table 1, we summarize a list of impeding behaviors reported on the managerial and engineering levels in related studies.

**Table 1.** Culturally distinct behaviors impeding agile ways of working.

Level	Impeding behavior	References
Management behavior	Command-and-control mindset, reinforced deference to superiors	[1, 5, 8, 10, 14, 30]
	Leadership style discouraging team members from exposing problems	[5]
	Leadership style discouraging from proposing alternatives to perceived directives from superiors	[5]
Engineers’ behavior	Willingness to say yes to most requests in deference to superiors, reluctance to warn about non-feasible deadlines	[1, 5, 30]
	Reluctance to expose problems	[1, 5, 8]
	Lack of commitment to self-learning, reliance on top-down improvements	[1, 30]
	Reluctance to engage in constructive disagreements and challenging discussions or voicing criticism	[8, 14, 15]
	Reluctance to propose alternatives to perceived directives from superiors	[5, 8]

The cited studies cover different countries within the Asian region, including India [5, 8, 27, 30], Malaysia and Singapore [1] and Asia Pacific in general [15], and are either based on interviews or own experiences.

A closer look at behaviors of engineers in India and neighboring countries reveals that most if not all impeding behaviors are likely to be caused by the hierarchical culture of the organizations and related management behavior, as suggested in related research [8, 29]. For example, Ayed et al. [1] report that Malaysian and Singapore engineers lacked the freedom to decide about their ways of working and therefore did not see the point in self-learning. But what if the hierarchical culture of command-and-control highlighted in numerous studies as poisonous to the agile ways of working [1, 5, 10, 14, 15, 30] would be replaced with the more empowering onshore management; would the offshore engineers working in mixed onshore-offshore teams be able to adopt the agile ways of working? Or would the less hierarchical Western companies fail to ignite the agile culture in their offshore collaborations? The answers to these questions are of high importance for shaping the understanding of the compatibility of agile ways of working with the use of offshoring.

## Research Methodology

To answer our research questions, we conducted an exploratory case study [24]. We executed our study in real world setting and studied collaboration between two companies (see Sect. 3.1). Our study is a holistic multiple-case study, where the context is the offshore collaboration between a mature agile company from Sweden and a hierarchical consultancy company from India; each case is a distributed agile team (five in our study) and individual behavior of the team members as the unit of analysis [24]. Finally, our data collection and analysis were divided into two steps (see Sect. 3.2).

### Empirical Background

The context of our study is a collaboration between an outsourcer/customer company from Sweden working in the telecommunication industry and an outsourcing vendor/a consultancy company from India. For confidentiality reasons we are prohibited to disclose the names of either of the companies. Our investigation targeted five distributed agile teams composed of team members from both the Swedish company and the offshore vendor from India. The selection of the teams was done with the help of the companies and represented all important business areas.

**Fig. 1.**
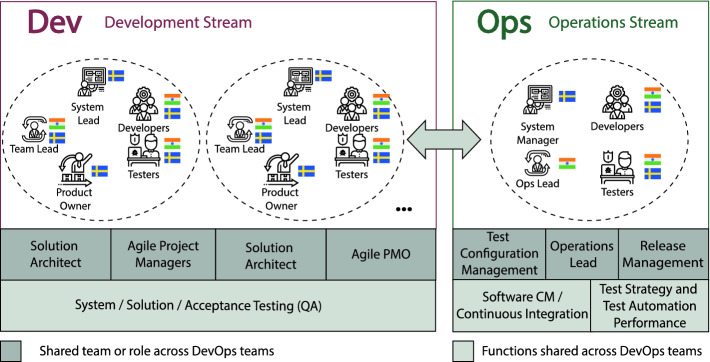
DevOps team structure

The studied teams were set up as DevOps teams consisting of a few smaller mixed Dev and Ops teams working accordingly in the development stream or the operation stream with supporting roles around. Each smaller team was cross-functional and involved developers, testers, a System lead, a Team lead and a Product owner (see the structure in Fig. 1 and profiles of the studied DevOps in Table 2).

**Table 2.** Profile of the studied teams.

	No of sites	Total no of members	No of participants	Participants		Offshore member roles
				Onshore	Offshore	
DevOps 1	2	18	18	11	7	Dev. (3), Test. (2), Architect, Op. lead
DevOps 2	2	22	20	14	6	Developers (5), Team Lead
DevOps 3	3	28	22	12	10	Dev. (6), Testers (2), Team leads (2)
DevOps 4	2	44	20	13	7	Developers (6), Operations Lead
DevOps 5	2	21	16	10	6	Consultant, Developers (4), Test lead
TOTAL		**133**	**96**	**60**	**36**	

DevOps teams followed agile principles and ways of working with iterative development (Scrum or Kanban, decided by each team individually), daily stand ups, and team retrospectives as the primary rituals. The offshore members of the teams were expected to follow the same agile principles and philosophy as the contracting organization. This is why cultural incompatibility across locations was seen as a threat.

### Data Collection and Analysis

**Fig. 2.**
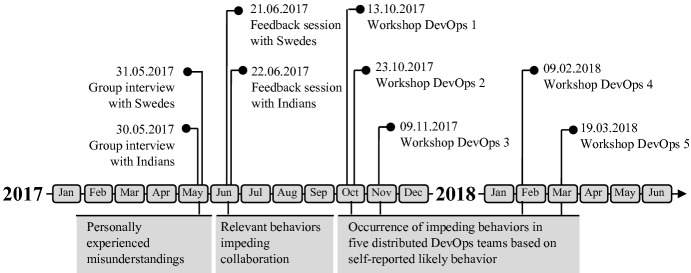
Data collection activities and resulting data on the timeline

A mixed approach was employed to study the impact of cultural differences on the collaboration between the Swedish customer and the Indian outsourcing vendor. Data collection was done in several steps including a quantitative data collection approach when exploring the personally experienced misunderstandings and relevant behaviors impeding collaboration, and a more qualitative approach when checking the occurrence of impeding behaviors in five distributed DevOps teams, as visualized in Fig. 2 and described in the following sub-sections.

#### Group Interviews to Elicit Misunderstandings.

First, we conducted separate homogeneous group interviews with representatives from Sweden and from India to elicit the main sources of misunderstandings that impede ways of working in the collaboration. Eight representatives participated in the session with the Swedish representatives (N = 8). Two experienced managers participated in the electronically mediated session with the Indian representatives (N = 2). The group interviews were conducted in May 2017, ran in English, and moderated by one of the researchers, while another researcher took detailed notes. Both sessions lasted approx. two hours and followed the same agenda – after getting to know each other and presenting the objectives, the participants were given time to connect to a web-based survey service called Mentimeter via mobile phones or computers and report personal experiences related to cultural misunderstandings. The survey form contained just one open question and the participants were encouraged to submit as many items as possible. The submitted items were then brought up one by one and discussed with all participants in the session. The situations in which certain misunderstandings occur were sought and every participant could add their own reflections and bring up new ideas, which were noted down as session notes.

The generated items and the recorded notes were analyzed in iterations. First, we aggregated elicited items in one list of misunderstandings grouped by similarity. This list contained seven larger categories related to cultural differences and their impacts. Then we revisited each category one by one, read through the session notes and formulated items in a particular form: *As a *<*role and/or site representative*>* it is confusing for me when *<*role and/or site representative*><*behavior*>* (when/in *<*situation*>*).*

#### Typical Impeding Behaviors Prioritized During Feedback Sessions.

The identified 26 different confusing behaviors served as the base for identifying situations, which were reported as sources of misunderstandings. To identify the most relevant impeding behaviors, we discussed our results in feedback sessions with larger groups of onshore and offshore representatives, conducted in June 2017. We discussed the identified problematic situations with expected and unwanted behavioral options in feedback sessions involving homogeneous groups of Swedish participants (N = 12) and Indian participants (N = 4), and refined our results based on the comments received. We also elicited the responses of the Swedish participants regarding the occurrence of the impeding behaviors on a scale: *Happens*, *Used to Happen*, *Never Happens*. This was not done in the session with Indian side representatives, because the respondents were too few and included a manager, who could have influenced the results. In this paper, we report a selection of behaviors that are classified as hindrances to the agile ways of working (see Sect. 4.1). These are based on the behaviors reported by both Indian and Swedish participants with the occurrence scores from the Swedish session. The behaviors that are not included in this paper included those related to the estimation precision, attitude towards time (reporting vacations, coming to meetings on time, extending work hours), communication (switching to local language), and a few variations of the behaviors.

#### Occurrence of Impeding Behaviors in Surveyed DevOps Teams.

We ran five workshops with DevOps teams (mixing onshore and offshore participants; N = 96) to discuss cultural differences and test the occurrence of impeding behaviors in each team (See the profiles in Table 2). The workshops were held in the fall of 2017 and spring of 2018. During the session we first queried the participants about their likely behavior in the given situations. Then each situation, reported behaviors and reasons for the likely behavior based on cultural studies [9] were discussed. At the end of each workshop, the areas of improvement for the participating team were identified. In this paper, we report and discuss the responses of offshore participants (N = 36) related to the behaviors impeding or enabling agile ways of working.

### Limitations and Threats to Validity

In this subsection, we discuss the limitations and issues that might threaten the validity of our results. Given the qualitative nature of our study, in the following we discuss the validity, reliability and generalizability threats following the guidelines by Leung [17].

**Validity** refers to the appropriateness of the method. We designed the empirical study in a two-staged fashion, aiming at improving objectivity when formulating and selecting the situations and impeding behaviors. We also used an anonymized data collection tool and obfuscated the results to ensure anonymity of the respondents, to eliminate the unwillingness to report personal confusions.

**Reliability** in qualitative research refers to the replicability of the results. A margin of variability in results is accepted when dealing with qualitative research [17] or mixed methods, since the subjectivity of the researcher is embedded in the roots of the analysis. The main threat is then related to consistency. To mitigate this threat, we let the participants report their responses in a data collection tool, by being systematic when taking notes and documenting the discussions during the sessions, and by keeping the quotations from the participants as exact as possible. Furthermore, we conducted feedback sessions to validate our interpretation of the impeding behaviors and situations.

Possible threats to the **validity** and **reliability** of our results are related to the reluctance of offshore participants to express their opinion in front of managers or onshore peers, as well as reluctance to talk about compromising issues. To alleviate these threats, we separated the Indian and Swedish participants in the initial group interviews and feedback sessions to be able to talk more openly. In the mixed workshop sessions, we explained the importance of truthful responses and asked onshore team leads to encourage openness. We also used a survey form that allowed participants to provide their responses anonymously, which remained untraceable to individuals even during the discussions. The reported problematic behavior in the survey, confirming comments received from the onshore participants and reflections voiced by more experienced offshore participants (in other words triangulation of the data sources) make us believe that we have elicited as honest and open responses as possible.

Data triangulation is the core principle of case study research [24]. To enhance the **validity** and **reliability** of our results, the individual responses elicited during the group interviews were first discussed in the respective groups and compared across the onshore and offshore groups. We then elicited quantitative data from larger groups of participants (survey responses) to minimize the bias towards selected individuals. The quantitative data elicited through the survey was further triangulated with the qualitative data (notes) from the discussions held during the workshops.

**Generalizability** of the conclusions drawn from our results are of course limited to the studied context. However, our results disprove an existing view that cultural barriers are likely to remain since the major differences in norms and values cannot be harmonized [14]. We believe that it is fair to assume that the gradual changes in behavior that we observed as a result of the gained experience with working in a mixed environment may also happen in similar contexts in other organizations.

## Results

In this section, we first list the culturally distinct behaviors that were reported to cause misunderstandings and impede the agile ways of working in distributed teams. Then we report on the occurrence of the impeding behavior from surveying 36 offshore members from a more hierarchical organization integrated into five DevOps teams.

### Behavior Impeding Agile Ways of Working

Based on the interviews, we identified 19 sources of misunderstandings reported by the Swedish participants and 14 sources of misunderstandings reported by the Indian participants. Notably, both sides reported what was confusing in the behavior of their counterparts as well as the own behavior that have led to misunderstandings or confusion. Our further analysis of the situations in which differences in behavior were seen as barriers for collaboration led to an aggregated list of 26 behaviors, which was triangulated with related literature. Of these, 12 behaviors were prioritized as frequently occurring and important to discuss as determined in the feedback sessions. In this paper, we provide an analysis of six of these behaviors that can be classified as impediments to agile ways of working (see Table 3).

**Table 3.**
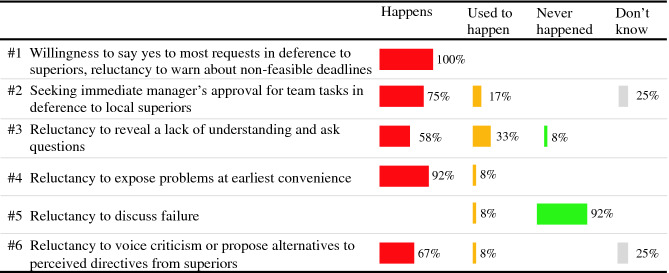
Behavior impeding agile ways of working and the frequency of occurrence reported by the Swedish representatives (N = 12).

The six reported behaviors impeding agile ways of working surface in the daily meetings, task allocation and content discussions, and team retrospectives; and appeared all but one as common sources of misunderstandings between the offshore and onshore members (see the column Happens in Table 3). Evidently, the most typical impediment is willingness to say yes to most requests in deference to superiors and reluctance to warn about non-feasible deadlines. Other impeding behaviors had varying frequency of occurrence. Some respondents indicated that although the impeding behaviors occurred in the past (“Used to happen” in Table 3), social integration of the Indian members led to the assimilation of the established ways of working and put an end to behavioral differences. This is why, in the next step we sought to further understand how common the impeding behaviors are in different teams, and what stimulates cultural integration.

### Behavior in Five Distributed Teams

Table 4 summarizes our results from surveying offshore members from five distributed DevOps teams regarding their likely behavior in six situations (impeding behaviors are emphasized in red color). Our results suggest that accepting unfeasible tasks in deference to superiors (#1), seeking immediate manager’s approval for tasks (#2) and reluctance to confess about lagging behind schedule (#4) are behaviors experienced in all five distributed teams, while confusing behavior in situations #5 and #6 are not that common. It is also evidently from the results that DevOps 1 and DevOps 5 appear quite successful with the cultural integration of their Indian members, while DevOps 2, 3 and 4 are challenged. This is confirmed by a more detailed analysis, which reveals that the most common respondents of impeding behavior are the recently onboarded members. In the following, we further detail our findings from analyzing survey responses and notes capturing the discussions the team members held during the sessions.

**Table 4.**
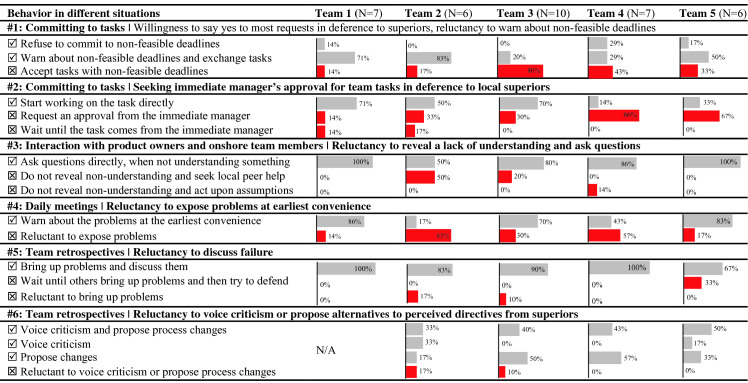
Self-reported offshore team member behavior in the given situations. Symbol  determines acceptable behavioral options, while  and the red color determines impeding behavioral options.

#### Willingness to Say Yes to Most Requests in Deference to Superiors, reluctance to warn about non-feasible deadlines.

This impeding behavior is one of the most common behaviors among our respondents, with eight members in DevOps 3 reporting to accept unfeasible tasks from superiors. As the technical product owner (TPO) from DevOps 3 reveals: *“We see [this impeding behavior] a lot, it does not just put us in a risk situation, but also other TPOs and stakeholders”*. The offshore member from Team 3 explained that it is difficult for them to say “No” and therefore they are likely to use hinting words as *“I will do my best”,* in the hope that it will be interpreted correctly. Yet, Swedes put no value on what is not said. Thus, team leads suggested that coaching offshore members to be direct and open was important. They explained that for Swedish team members, a “Yes” means “I understand”, “I agree”, “I accept”, “I approve”.

#### Seeking Immediate Manager’s Approval for Team Tasks in Deference to Local Superiors.

This challenge was seen as a fact of life, since Indian team members were a part of the consultancy company and confirmed to local rules and regulations. In fact, many team leaders and product owners established direct communication channels with offshore managers. Therefore, this behavior was not seen as a major impediment. However, the leader of DevOps 2 was recently employed and appeared to be unaware of this difference in behavior, perhaps because a lack of onboarding into the cultural norms of the team. This lack of awareness was seen as problematic since she did not know of the importance of maintaining regular communication with the offshore managers.

#### Reluctance to Reveal a Lack of Understanding and Ask Questions.

Few members of DevOps 2 and 3 will hide a lack of understanding when discussing requirements with the product owner, and instead ask peers for help, while one respondent in Team 4 will proceed based on the best assumptions. Members of DevOps 1 discussed that they experienced this challenge especially among the new Indian members joining the team and that exchange visits and personal acquittance between the product owner and the offshore members helps. Interestingly, an offshore member from DevOps 3 said to be surprised by the results and that from his observation, people are still reluctant to ask questions. He explained: *“…when we have discussion between sites, I can see that an offshore person doesn’t understand, but doesn’t really say that. They might search for an answer in the meantime and then come back with the answer, but probably won’t say it in meeting”.* Product owner in DevOps 3 suggested that one way to promote the wanted behavior is to encourage questions; he explained: *“I am used to say if you don’t have questions, you don’t understand”*. Another useful advice was the “Talk back” approach, which suggests not to ask Yes/No questions, but rather ask to summarize what was said or agreed, or to explain the next steps one would do after the discussion.

#### Reluctance to Expose Problems at Earliest Convenience.

Another common challenge in all five teams was the fear of revealing the fact that someone is lagging behind during team daily meetings. As an offshore member of DevOps 1 explained: *“Everyone wants to do their best, they try everything before saying that they are late”*. The reluctance to expose problems was linked to the deference to managers in typically hierarchical organizations and the “Why-management” style (A Why? Question follows when someone reveals a problem, forcing people to engage in uncomfortable explanations and thus making them “lose face”). The meetings were said to differ in Sweden and in India. An offshore member explained: *“They have in mind that the boss is the one who decides their salary”,* while the team leads and managers in Sweden over time were accepted as more accessible and thus raising problems became less frightening. Swedish members emphasized that they prefer engineers to say what they think, and not what they think managers want them to say.

#### Reluctance to Discuss Failure.

This challenge was not reported as common in the studied teams, which was motivated by the positive atmosphere of the retrospective meetings with the Swedish team leads. An offshore member of DevOps 1 further explained: *“It depends on who is in the meeting, sometimes we keep silent. It depends who is asking, and who is running the meeting”*. Therefore, keeping local offshore managers outside the retrospectives was seen as an important learning.

#### Reluctance to Voice Criticism or Propose Alternatives to Perceived Directives From Superiors.

Similarly, challenging the established ways of working during the retrospectives did not appear as a common challenge. Many offshore members were likely to either challenge the processes or propose improvements.

## Discussion

### Cultural Barriers Impeding the Agile Ways of Working

In response to the first research question, we have identified several behaviors rooted in the hierarchical forms of organization as impediments for agile ways of working, which confirm previous research that highlight cultural barriers common to offshore organizations. In particular, we found the reinforcement of deference to superiors [5, 8, 14] to be a common barrier for the studied teams. This in our case led to a willingness to say yes to even unrealistic requests (similarly to [1, 5, 30]), reluctance to expose problems (similarly to [1, 5, 8, 30]) and reluctance to reveal the lack of understanding and ask questions to a superior. At the same time, our findings suggest that the empowering culture and democratic leadership from Sweden encourage the trust and transparency-based behavior among the offshore members. However, the behavioral transformations take time and cannot be taken for granted. In the presence of hierarchical structures and command-and-control leadership locally, cultural integration might take longer, or result in that offshore team members are forced to assimilate different behaviors in parallel. In fact, during one of the workshops an offshore team lead told us that offshore engineers that move between customers might not be rewarded for what is a typical behavior in an agile environment and need to revert to the behaviors typical to hierarchical cultures.

When contrasting our findings with the organizational culture factors that correlate with effective use of an agile method [8, 29], we can say that the culture of the outsourcing vendor did not confirm with the highlighted values and management style. The implication of this is that agile companies might be more likely to succeed with offshoring through establishing their own sites; working with vendors that are already used to agile values and ways of working or recruiting people that matched the culture of the existing teams [22]. In particular, the companies may want to assess the vendors management style, i.e., facilitation-leadership, collaboration-oriented management style and focus on empowerment as crucial factors for adopting agility [29].

### Cultural Integration of Offshore Members from a Non-agile Organization

Unlike researchers who suggest that cultural barriers are likely to remain since the major differences in norms and values cannot be harmonized, as they derive from deep-seated differences in cultural background, education, and working life [14], we found that behavior seem to change when engineers are exposed to the culturally distinct environment. Our findings demonstrate that Swedish members succeeded in integrating members of a hierarchical culture and stimulating the changes in their behavior. Our findings are in accordance with Iivari et al. that found that the relationship between an organizational culture and agile ways of working is dynamic [12]. Our experience, however, shows that distributed teams are often left to experiment and adjust their ways of working, since cultural awareness is often gained with experience. This has been noted by Casey [3], who found that the importance of and requirement for cultural training is often not recognized before a lot of time, effort and resources are wasted. We therefore recommend companies and distributed teams to run cross-cultural communication courses to discuss the values that should govern behavior as also suggested in [16]. For team leads and onshore managers struggling to overcome the same cultural barriers as reported in this article, we can recommend the following:

- Establish good communication channels with local offshore managers and agree on an efficient task allocation procedure;
- Schedule more frequent check-ins or updates;
- Create an environment of psychological safety and trust; set an example of taking ownership for failures safely; show how teams can learn from mistakes, reward people for making mistakes if/when they lead to valuable lessons;
- Seek out *one*-*to*-*one* conversations; encourage offshore members to be more direct, use “Talk Back” approach to check the understanding;
- Encourage suggestions of better ways of working; do not criticize ideas; complement people for valuable input; set an example of changes that led to action;
- Avoid offshore managers’ presence in team retrospectives and meetings where honest and open input and feedback from offshore members is important.

## Conclusions

Distributed collaboration is challenging [10]. In this paper, we explore one specific challenge, i.e., dealing with barriers rooted in the differences in national and organizational cultures. Our results confirm the existing research that organizational and national cultural barriers may impede collaboration in general and adoption of agile ways of working in particular. However, our empirical findings from studying behavior of offshore members from five mixed DevOps teams suggest that behavior does change over time, even when integrating offshore engineers who are used to radically different hierarchical organizational culture into agile ways of working. In other words, Indian developers working in agile projects, learned how to do as their more experienced agile team members from Sweden do. Our study resulted in a list of recommendations for companies willing to discuss cultural differences and foster cultural integration in offshore projects.

## References

[1] Ayed, H., Vanderose, B., Habra, N.: Agile cultural challenges in Europe and Asia: insights from practitioners. In: IEEE/ACM 39th International Conference on Software Engineering: Software Engineering in Practice Track (ICSE-SEIP), pp. 153–162 (2017)

[2] Baruah N, Ashima A (2012). A survey of the use of agile methodologies in different Indian Small and Medium Scale Enterprises (SMEs). Int. J. Comput. Appl..

[3] Carmel E, Agarwal R (2001). Tactical approaches for alleviating distance in global software development. IEEE Softw..

[4] Casey, V.: Leveraging or exploiting cultural difference? In: 2009 Fourth IEEE International Conference on Global Software Engineering, pp. 8–17 (2009)

[5] Fowler, M.: Using an agile software process with offshore development. https://www.martinfowler.com/articles/agileOffshore.html

[6] Gallivan M, Srite M (2005). Information technology and culture: Identifying fragmentary and holistic perspectives of culture. Inf. Organ..

[7] Hall, E.: The Silent Language. Doubleday, Garden City (1959)

[8] Hoda, R., Noble, J., Marshall, S.: Supporting self-organizing agile teams. In: International Conference on Agile Software Development, Madrid, Spain, pp. 73–87 (2011)

[9] Hofstede G, Hofstede GJ, Minkov M (2010). Cultures and Organizations.

[10] Holmström H, Fitzgerald B, Ågerfalk PJ, Conchúir EÓ (2006). Agile practices reduce distance in global software development. Inf. Syst. Manag..

[11] Iivari, J., Iivari, N.: The relationship between organizational culture and the deployment of agile methods. In: Information and Software Technology, pp. 509–520. Elsevier (2011)

[12] Iivari N (2010). Culturally compatible usability work: an interpretive case study on the relationship between usability work and its cultural context in software product development organizations. J. Organ. End User Comput..

[13] Kautz, K., Pedersen, C.F., Monrad, O.: Cultures of agility - agile software development in practice. In: ACIS 2009 Proceedings - 20th Australasian Conference on Information Systems, pp. 174–184 (2009)

[14] Krishna S, Sahay S, Walsham G, Hirschheim R, Heinzl A, Dibbern J (2006). Managing cross-cultural issues in global software outsourcing. Information Systems Outsourcing (Second Edition): Enduring Themes.

[15] Lee S, Yong HS (2010). Distributed agile: project management in a global environment. Empir. Softw. Eng..

[16] Lenberg P, Feldt R, Wallgren Tengberg LG (2019). Misaligned values in software engineering organizations. J. Softw. Evol. Process..

[17] Leung L (2015). Validity, reliability, and generalizability in qualitative research. J. Fam. Med. Prim. care..

[18] Van Maanen, J., Laurent, A.: The flow of culture: some notes on globalization and the multinational corporation*. In: Organization Theory and the Multinational Corporation, pp. 275–312. Palgrave Macmillan UK (1993)

[19] Moe NB, Dingsøyr T, Dybå T (2010). A teamwork model for understanding an agile team: a case study of a Scrum project. Inf. Softw. Technol..

[20] Moe NB, Baumeister H, Weber B (2013). Key challenges of improving agile teamwork. Agile Processes in Software Engineering and Extreme Programming.

[21] Moe, N.B., Dahl, B., Stray, V., Karlsen, L.S., Schjødt-Osmo, S.: Team autonomy in large-scale agile. In: Proceedings of the Hawaii International Conference on System Sciences (2019)

[22] Moe, N.B., Stray, V., Goplen, M.R.: Studying onboarding in distributed software teams: a case study and guidelines. In: Evaluation and Assessment in Software Engineering, April 15–17, 2020, Trondheim, Norway. ACM, New York (2020)

[23] Morgan G (2006). Images of Organization.

[24] Runeson P, Host M, Rainer A, Regnell B (2012). Case Study Research in Software Engineering.

[25] Šmite D, Moe NB, Ågerfalk PJ, Šmite D, Moe N, Ågerfalk P (2010). Fundamentals of agile distributed software development. Agility Across Time and Space.

[26] van Solingen R, Berghout E, Kusters R, Trienekens J (2000). From process improvement to people improvement: Enabling learning in software development. Inf. Softw. Technol..

[27] Srinivasan, J., Lundqvist, K.: Agile in India: challenges and lessons learned. In: Proceedings of the India Software Engineering Conference, pp. 125–130. ACM Press (2010)

[28] Stray V, Fægri TE, Moe NB, Abrahamsson P, Jedlitschka A, Nguyen Duc A, Felderer M, Amasaki S, Mikkonen T (2016). Exploring norms in agile software teams. Product-Focused Software Process Improvement.

[29] Strode, D.E., Huff, S.L., Tretiakov, A.: The impact of organizational culture on agile method use. In: Proceedings of the Annual Hawaii International Conference on System Sciences, pp. 1–9 (2009)

[30] Summers, M.: Insights into an Agile adventure with offshore partners. In: Proceedings - Agile 2008 Conference, pp. 333–338 (2008)

[31] Williams L (2012). What agile teams think of agile principles. Communications of the ACM..

[32] Agile India 2012 survey results final. https://www.slideshare.net/ivaltech/agile-india-2012-survey-results-final. Accessed 11 Feb 2020

